# Knowledge of Future Dental Practitioners towards Oral Cancer: Exploratory Findings from a Public University in Malaysia

**DOI:** 10.1155/2015/218065

**Published:** 2015-12-29

**Authors:** Akshaya Srikanth Bhagavathula, Nazrin Bin Zakaria, Shazia Qasim Jamshed

**Affiliations:** ^1^Department of Clinical Pharmacy, University of Gondar, College of Medicine and Health Sciences, School of Pharmacy, Gondar, Ethiopia; ^2^Department of Pharmacy Practice, Kulliyyah of Pharmacy, International Islamic University Malaysia, Kuantan, Pahang, Malaysia

## Abstract

*Objective*. To assess knowledge and awareness of oral cancer in the early identification of risk factors among undergraduate dental students.* Methods*. A total of 162 undergraduate (third, fourth, and fifth year) dental students at International Islamic University, Malaysia, were approached to participate in the study, and those who agreed were administered. A 9-item pretested questionnaire contains questions on oral examination, oral cancer risk factors, and requests for further information. Descriptive statistics were conducted using chi-square testing.* Results.* The response rate of the study was 70.3% (114/162), with 26 (22.8%) males and 88 (77.2%) females. All undergraduate dental students were familiar with examining the oral mucosa of their patients and most were likely to advise patients about the risk factors for developing oral cancer (98.2%). Nearly one-third (32.4%) of students reported examining patients with oral lesions as early signs for oral cancer (*P* < 0.001) and nearly 70% agreed that they did not have sufficient knowledge regarding the prevention and detection of oral cancer (*P* < 0.001). In addition, more than 95.6% agreed that there is a need for additional information/teaching regarding oral cancer. Further, 61.3% and 14.1% identified tobacco smoking and drinking alcohol as major risk factors for developing oral cancer.* Conclusion.* This study demonstrated lack of awareness about risk factors among undergraduate dental students regarding oral cancer. Reinforcing awareness and enhancing the benefits of early detection on prevention of oral cancer should be done through training and/or educational intervention.

## 1. Introduction

The incidence of oral cancer especially squamous cell carcinoma accounts for nearly 2.4% of all cancers [1]. Due to significant number of oral cancer cases raising rapidly in the developing regions this is found to be the sixth most common cancer worldwide [2]. Life style habits such as heavy smoking and alcoholism are the important risk factors for developing oral cancer that increases at least three- to fifteenfold especially in females and young people [3]. In addition, marijuana, chewing beetle-leaf, human papilloma virus, ultraviolet radiations, iron deficiency anemia, candida infections, immunosuppression, and deletion or mutation of tumor suppressor genes are some of the other causes of oral cancer [4].

Lack of public awareness regarding oral health and low intake of fruits and vegetables, older age, and poor oral hygiene are some of the implications for oral cancer [2]. Majority of the oral cancer was detected at late stages (III and IV) and early diagnosis is important to increase patient survivability and to delay its prognosis [5]. In 2011, World Health Organization (WHO) reported the incidence of oral cancer deaths in Malaysia to about 1.5% of the total deaths, with age adjusted death rate of 7.72 per 100,000 populations [6]. Malaysia ranked 14 in the world with annual oral cancer deaths of 1,587 [7].

Increasing the public awareness and early diagnosis can significantly improve oral cancer surveillance and prevent the delaying factors. To achieve these, it is important to have sufficient knowledge and awareness among dentists for detection and early diagnosis. Initiatives were undertaken by University of Malaya to increase the oral cancer awareness in Malaysia such as Malaysian Oral Cancer Research Initiative (MOCRI) and Oral Cancer Research & Coordinating Centre (OCRCC) [8]. These publicity initiatives are crucial to improve the oral cancer awareness among general public and health professionals in Malaysia. In addition, general dental practitioner's role is decisive in identifying the oral mucosal changes that may lead to oral cancer. Assessing the knowledge of dental students paves the way towards understanding their level of awareness in the early detection and prevention of oral cancer. To the best of our knowledge, previous researches on dental students' knowledge and awareness were conducted in the University of Malaya (UM) and Universiti Sains Malaysia (USM) [9, 10]. Since there is a paucity of information regarding oral cancer awareness in undergraduate dental students in different other regions of Malaysia, therefore it is pertinent to assess these characteristics in senior dental students (third, fourth, and fifth year) at International Islamic University, Kuantan, Pahang, Malaysia. The aim of the current research was to assess the knowledge and awareness of oral cancer towards early identification of risk factors among undergraduate dental students.

## 2. Methodology

This is a descriptive cross-sectional study to assess the oral cancer knowledge and awareness of senior undergraduate dental students using a survey questionnaire, adopted by Carter and Ogden [11] and Brzak et al. [12]. Ethical permission to conduct the study was obtained from the respective Deans, International Islamic University, Malaysia. The study was conducted via face-to-face interview at International Islamic University Malaysia during the period of February to March, 2015. Verbal consent was obtained from each participant prior to administration of study questionnaire.

### 2.1. Sampling Technique

Sample size was determined using 95% confidence interval, with an accuracy of 60% for the total dental students being 300 studying in International Islamic University given a confidence interval of 5.5; the recommended sample size is 155 or more. A systemic random sampling technique was used to select senior dental students which includes third, fourth, and fifth years.

In general, as included in the curriculum, dental students receive information regarding oral cancer during their oral pathology and oral medicine sessions in their first and second year as well as oral examination during their clinical sessions. Students of both gender studying third-, fourth-, and fifth-year dentistry were included in the study. Students who failed to meet the above criteria were excluded.

A 9-item pretested questionnaire was employed after explaining the purpose of the study and verbal consent was obtained from each study participant. The questionnaire constitutes 7 close-ended (yes/no) questions such as (1) oral mucosal examination (2 items), (2) advising current and future patients regarding risk factors for oral cancer, (3) opportunity to examine oral lesions, (4) knowledge regarding prevention and detection of oral cancer, (5) point of referral selection, and (6) desire for further information or teaching regarding oral cancer. Two open-ended questionnaires were asked to identify the risk factors for development of oral cancer and encouraged to select at least three to four options out of 10 options. In addition, interest of preferences for obtaining oral cancer information (1 out of 3 options).

Statistical analysis was performed using SPSS version 21 (SPSS Inc., Chicago, IL, USA). Descriptive data were analysed using frequencies and percentages. The Wilcoxon rank-sum test and chi-square were used to identify the difference between groups. The level of significance was set at *P* < 0.05.

## 3. Results

A total of 162 students were approached, and 114 questionnaires were returned with an overall response rate of 70.3%. Eighty-eight were females and twenty-six were males with a mean age (standard deviation) of 24.36 (7.12). Sex distribution with the number of respondents per year of course was shown in Table 1.

## 4. Knowledge about Oral Cancer

**Figure 1:**
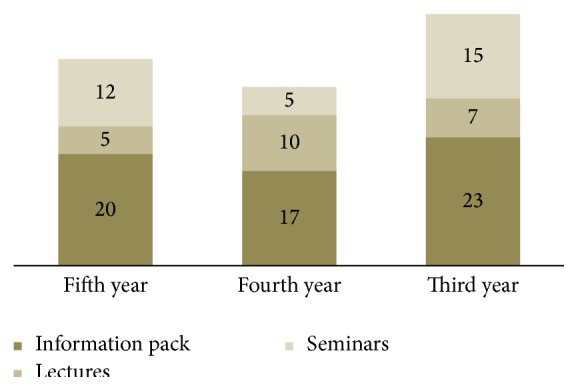
Students preferences for obtaining further information about oral cancer.

When asked about the examination of oral mucosa of the patients, all the students answered “yes” during their clinical training. Of those who examine the oral mucosa routinely, a high majority of the students (97.3%) would not examine the oral mucosa of the patient with high risk of developing oral cancer. Significantly, 67.5% of the students did not get opportunity to examine the oral lesions (*χ* ^2^ = 15.892, df = 2, and *P* = 0.000) (Table 2). More than ninety percent of the participants preferred to refer patients with oral lesion as a point of care to dental specialties rather than doctors. However, significantly, two-thirds (65.7%) of students felt that they did not have sufficient knowledge about prevention and early detection of oral cancer. This was much higher observed in third-year student participants (42/45) than others (*χ* ^2^ = 28.598, df = 2, and *P* = 0.000). Of note, most of the study participants (95.6%) requested further information regarding oral cancer prevention and early detection, with more than fifty percent preferred to obtain in the form of information package (52.6%), twenty-eight percent through seminars, and nearly twenty percent as lectures (Figure 1).

## 5. Risk Factors for Oral Cancer

**Figure 2:**
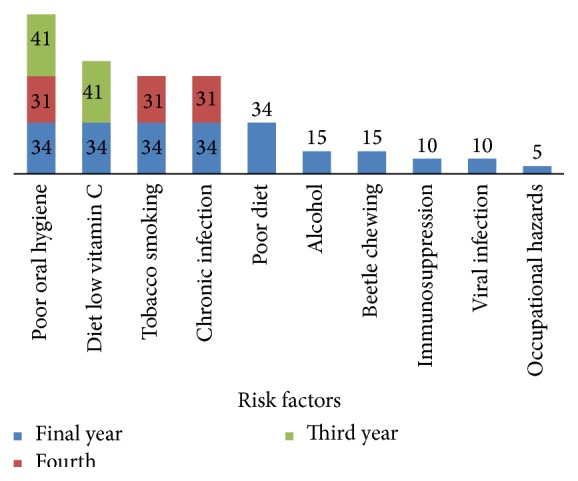
Dental students identifying risk factors for oral cancer (*N* = 106).

A majority of the dental students (93%) identified a number of different risk factors for oral cancer were shown in Figure 2. All the participants identified poor oral hygiene as a major risk factor, whereas 70.7% identified diet with low vitamin c levels as a risk factor for oral cancer. In addition, tobacco smoking and chronic infections were correctly reported by 61.3%. Only 14.5% of the participants identified alcohol and chewing beetle leaves as a risk factors. However, other oral cancer risk factors such as immunosuppression, viral infections, and occupational hazards were poorly reported by the final year students, and none of the other students identified these as a risk factors.

## 6. Discussion

Squamous cell carcinoma accounts for 90% of oral cancer and it is a general practice of the dental students to examine the patients' oral mucosa. Providing opportunity to examine and early detection can reduce the morbidity of oral cancer especially in high risk patients. It is the prime responsibility of the dental schools to provide sufficient knowledge to students for early diagnose in asymptomatic patients and prevent prevalent oral diseases. Hence, this study was carried out to determine the level of knowledge and awareness of oral cancer among dental students at International Islamic University, Selangor, Malaysia.

The response rate of the current study was 70.3% which is much lower than the studies conducted on dental students in Croatia (95%) [12], India (90.6%) [13], Iran (88%) [1], and Brazil (75.1%) [14] but fairly higher than the similar studies conducted in other specialties [15]. Although the response rate was low, a comparable number of students from different academic years participated in the study.

### 6.1. Level of Knowledge

In the present study, all the student participants routinely carried out oral mucosal examination of patients. Further, it was identified that a large majority of the students had an opportunity to examine patients with oral lesions. But unfortunately, a high majority of these students claimed that they failed to screen the high risk patient groups, which implies the gaps in their knowledge regarding oral cancer risk factors. Poor knowledge is directly related to lack of awareness, and emphasis should be taken to provide more opportunities engaging undergraduates to take oral health histories and examine oral lesions in patients during clinical attachments that should be undertaken. It is arguable that majority of the oral cancer patients are asymptomatic and identifying the changes in their cancerous and precancerous lesions in the oral cavity would help them to apply their critical knowledge into practice, importantly needed in high prevalent countries like Malaysia. For such reasons, Ogden et al. [1] claimed to implement work-based assessments to know these gaps and specific test for oral cancer within the curriculum prior to dental students graduation.

Regarding referral pattern for oral cancer, more than ninety percent felt that it is the dentist's responsibility to diagnose the oral malignancies. These results are encouraging as they demonstrate the recognition of dentistry, and it is their responsibility of dentists to diagnose and evaluate oral cancer. These results were consistent with other studies conducted by Ogden and Mahboobi [1], Awan et al. [9], Carter and Ogden [11], and Fotedar et al. [13] but contradict with study by Brzak et al. [12] where majority of the undergraduate dental students chose to refer oral cancer patients to a plastic surgeon specialist. A recent meta-analysis concluded that diagnosis delay is a potential risk factor for developing advanced stage oral cancer [16].

In our study, the majority reported that they would advise their patients about oral cancer and associated risk factors after graduation. These findings were similar to the previous study performed in Malaysia [9], UK [10], and Croatia [12]. It is crucial role of dentists to take a strong responsibility to offer advice to the patients on high-risk habits like cessation of smoking cigarettes [17] and self-examination of oral mucosa to improve the oral hygiene. These counseling techniques also enhance early detection of oral mucosal changes in the oral cavity [9].

Approximately, seventy percent felt that they are have insufficient knowledge (*P* < 0.001) with regard to prevention and early detection of oral cancer. These numbers are higher in those who were in third year (36.8%). In McCready et al.'s study [15] 77% of dental students from second year and fourth year reported that they were poorly informed regarding oral cancer, whereas in Carter and Ogden's study [11] 93% of the final-year medical students also reported the same. A well-designed institutional-based clinical training by incorporating different dental specialties such as oral medicine, dental oncology, and oral and maxillofacial surgery to improve the knowledge about oral cancer is highly recommended to increase the students confidence. However, almost all the students requesting further information regarding oral cancer which is similarly identified in studies by Awan et al [9], Brzak et al [12], and McCready et al [15] where more than 90% of the students requested to receive more information regarding oral cancer. Further, majority of the students are interested in receiving further information in the form of information package which is also most preferred in other studies [1, 9, 10, 12]. Seoane et al. [18] study assessing the oral cancer prevention and clinical attitude among Spanish dentists highlighted that providing continuous education through scientific newsletters and journals can provide positive preventive attitude in oral cancer.

### 6.2. Risk Factors for Development of Oral Cancer

In our study, 106 out of 114 participants identified the risk factors for oral cancer. Of these, all the students felt poor oral hygiene as the single most important risk factor. Only sixty percent of students identified tobacco smoking as a risk factor for development of oral cancer. Although previous studies revealed that smoking tobacco and alcohol consumption increase the incidence of oral cancer, these were unidentified by our third-year dental students. These findings were contradictory with other studies in the literatures, which show that around 90% of the dental and medical students identified tobacco smoking as an important risk factor 12,58 [1, 9, 10, 12–14, 17, 19]. However, alarmingly, none of the third and fourth years identified alcohol, beetle-chewing, and immunosuppression as a risk factor. Thus the knowledge on risk factors was poor in both third and fourth years and also very minimal in final-year dental students. There was trend towards better identification of risk factors which was observed with progression of their academic years, which is similarly noticed in other studies [9, 11, 14]. All these findings identified different knowledge gaps in identification of risk factors among dental students, and there is a need of educational intervention by implementing training or workshop particularly focusing on oral cancer.

## 7. Study Limitations

We used a prevalidated questionnaire which is used in other surveys assessing oral cancer knowledge [9, 11] on dental students to reduce selection bias. Further, recent research identified that nearly 20–30% of the oropharyngeal squamous cell cancer did not have traditional risk factors of smoking and cancer [20] which may be falsely interpreted in the light of respondents' knowledge.

The study has some limitations that should be taken into consideration. The study was conducted on senior dental students in a single institution in Malaysia and may not be generalized to other regions. In addition, the data presented here is self-reported, and some of the respondents may provide extreme responses than others, due to the motivations and beliefs of the participants, and might be subjected to recall bias. However, we believed that the participants were honest to provide appropriate responses conducted in a single institution, and national level multifaceted studies are further needed to assess dental students' knowledge about oral cancer.

## 8. Conclusion

This study showed knowledge gaps about oral cancer among undergraduate dental students. Lack of awareness about the risk factors initiates the need based educational interventions among future dental practitioners regarding early detection and prevention of oral cancer in Malaysia.

## Figures and Tables

**Table 1:** Age and sex distribution of dental student respondents.

Number of students approached		Student participants (%)		Male	Female
Total	162	114 (70.3)		26 (22.8%)	88 (77.2%)
Fifth year	52	37 (32.5)		12 (10.5%)	25 (21.9%)
Fourth year	40	32 (28.1)		6 (5.2%)	26 (22.8%)
Third year	70	45 (39.5)		8 (7%)	37 (32.4%)

Participants age	Mean	Standard deviation	Median	IQR^*∗*^	Wilcoxon rank-sum test

	24.36	7.127	24	1	*P* < 0.001

^*∗*^IQR: interquartile range.

**Table 2:** Level of knowledge of participants on oral cancer.

Variables	Dental students (%)			Total 114 (%)	Stat. cal. value
	Fifth year (%)	Fourth year (%)	Third year (%)		
Do you examine patients' oral mucosa routinely?					—
Yes	37 (32.4)	32 (28.0)	45 (39.4)	114 (100%)	
No	0	0	0	0 (0.0)	
Do you screen the oral mucosa if the patients are in high risk of categories?					*χ* ^2^ = 2.358, DF = 2, *P* = 0.308
Yes	2 (1.7)	1 (0.8)	0	3 (2.6)	
No	35 (30.7)	31 (27.2)	45 (39.4)	111 (97.3)	
When you have graduated will you advise patients about the risk factors for oral cancer?					*χ* ^2^ = 0.822, DF = 2, *P* = 0.663
Yes	36 (31.5)	32 (28.0)	44 (38.6)	112 (98.2)	
No	1 (0.8)	0	1 (0.8)	2 (1.7)	
Have you had the opportunity to examine patients with oral lesions?					*χ* ^2^ = 15.892, DF = 2, ***P* = 0.000** ^*∗*^
Yes	13 (11.4)	18 (15.7)	6 (5.2)	37 (32.4)	
No	24 (21.0)	14 (12.2)	39 (34.2)	77 (67.5)	
Do you think a patient should go to a doctor or dentist if he/she has an oral lesions?					*χ* ^2^ = 2.107, DF = 2, *P* = 0.349
Doctor	5 (4.3)	3 (2.6)	2 (1.7)	10 (8.7)	
Dentist	32 (28.0)	29 (25.4)	43 (37.7)	104 (91.2)	
Do you feel that you have sufficient knowledge concerning prevention and detection of oral cancer?					*χ* ^2^ = 28.598, DF = 2, ***P* = 0.000** ^*∗*^
Yes	23 (20.1)	13 (11.4)	3 (2.6)	39 (34.2)	
No	14 (12.2)	19 (16.6)	42 (36.8)	75 (65.7)	
Would you like more information or teaching on oral cancer?					*χ* ^2^ = 4.055, DF = 2, *P* = 0.132
Yes	35 (30.7)	29 (25.4)	45 (39.4)	109 (95.6)	
No	2 (1.7)	3 (2.6)	0	5 (4.3)	

^*∗*^Significant at *P* < 0.05 were bold.
